# A comparative study of dexmedetomidine versus clonidine as additives for spinal anesthesia: A meta-analysis of clinical trials

**DOI:** 10.1097/MD.0000000000048102

**Published:** 2026-03-20

**Authors:** Meijuan Yang, Yu Zhang, Zhiyou Peng

**Affiliations:** a Department of Anesthesiology, Women’s Hospital, Zhejiang University School of Medicine, Hangzhou, Zhejiang, China; b Department of Painology, The First Affiliated Hospital, Zhejiang University School of Medicine, Hangzhou, Zhejiang, China.

**Keywords:** clonidine, dexmedetomidine, meta-analysis, spinal anesthesia

## Abstract

**Background:**

To evaluate the efficacy of dexmedetomidine versus clonidine as adjuvants to local anesthetics in spinal anesthesia.

**Methods:**

We searched PubMed, Embase, Web of Science, and the Cochrane Library databases for randomized controlled trials (RCTs) comparing the effects of dexmedetomidine and clonidine when used as adjuvants to intrathecal local anesthetics.

**Results:**

A total of 10 randomized controlled trials were included in this meta-analysis. Compared with clonidine, dexmedetomidine significantly improved spinal block characteristics, including a faster onset of sensory block (standardized mean difference: −0.57, 95% confidence interval [CI]: −1.06 to −0.090; *P* < .05), a longer duration of sensory block (mean difference [MD] = 29.87 minutes, 95% CI: 21.89–37.86; *P* < .05), a longer duration of motor block (MD = 31.12 minutes, 95% CI: 14.51–47.73; *P* < .05), and a prolonged time to first rescue analgesia (MD = 21.86 minutes, 95% CI: 13.14–30.59; *P* < .05). No statistically significant differences were observed between the clonidine and dexmedetomidine groups with respect to the incidence of nausea and vomiting, bradycardia, hypotension, or shivering (*P* ≥ .05).

**Conclusion:**

When used as an intrathecal adjuvant and compared with clonidine, dexmedetomidine yields superior spinal block characteristics, including a shorter onset time of sensory block, longer durations of both sensory and motor block, and an extended time to first rescue analgesia. No differences were detected between the 2 drugs in the incidence of nausea and vomiting, bradycardia, hypotension, and shivering.

## 1. Introduction

Prolongation of sensory block duration and optimization of postoperative pain relief are core concerns for both anesthesiologists and patients undergoing surgical procedures under intrathecal anesthesia.^[1]^ α_2_-adrenergic agonists have been proven to possess multiple beneficial properties in the context of local anesthesia, such as potentiating the anesthetic effect, reducing the required dosage of local anesthetics, mitigating intraoperative hemodynamic fluctuations, and alleviating postoperative pain.^[2,3]^ Owing to these advantages, they have become a hot topic in research on local anesthetic adjuvants in recent years.

Dexmedetomidine, a highly potent and selective α_2_-adrenergic agonist, and clonidine, a partial selective α_2_-adrenergic agonist, share similarities in their clinical application profiles and safety profiles.^[4,5]^ Compared with clonidine, dexmedetomidine has a significantly higher affinity for α_2_ receptors - its affinity for α_2_ receptors is approximately 8 times that of clonidine. Moreover, it has an extremely low affinity for α_1_ receptors, with an α_2_/α_1_ selectivity ratio of 1600:1, which is much higher than that of clonidine (200:1). This high selectivity enables dexmedetomidine to exert more potent sedative and analgesic effects while minimizing the incidence of adverse reactions such as hypotension and bradycardia associated with α_1_ receptor activation.

Studies comparing the efficacy and safety of clonidine versus dexmedetomidine when used as adjuvants to intrathecal local anesthetics (e.g., bupivacaine or ropivacaine) have yielded conflicting results.^[6–8]^ Some research suggests that dexmedetomidine may prolong sensory or motor block duration, while other studies report no significant differences between the 2 drugs in these outcomes. Therefore, the present study aims to compare the efficacy of dexmedetomidine and clonidine as adjuvants to local anesthetics in spinal anesthesia.

## 2. Methods

### 2.1. Search strategy

The present meta-analysis was conducted in accordance with the recommendations outlined in the Preferred Reporting Items for Systematic Reviews and Meta-Analyses statement and the Cochrane Handbook for Systematic Reviews of Interventions.^[9,10]^ The protocol was registered with PROSPERO (Registration number: CRD420251137403). Ethical approval was not required for this meta-analysis, as it is based on previously published studies. All data analyzed are publicly available in the existing literature, and no original human participants or animals were involved. Therefore, informed consent was not applicable.

Two independent authors (Meijuan Yang and Yu Zhang) systematically searched PubMed, Embase, Web of science, and Cochrane Library databases up to July 15, 2025. To ensure no eligible studies were overlooked, we further manually screened the reference lists of all included primary studies and key relevant systematic reviews to identify additional literature meeting our predefined inclusion criteria. The search strategy combined free-text terms and Medical Subject Headings terms, including dexmedetomidine, clonidine, intrathecal, and randomized controlled trial. The PubMed search strategy was adapted for each database. Only human studies published in English and Chinese were included.

### 2.2. Inclusion criteria

Studies meeting the following criteria were included for further analysis: original, independent studies; randomized controlled trials (RCTs); Intervention and comparator: Adult patients receiving intrathecal dexmedetomidine or clonidine (as the intervention or comparator, respectively).

### 2.3. Exclusion criteria

Studies meeting any of the following criteria were excluded: nonrandomized controlled trials (nonRCTs), systematic reviews, conference abstracts, preclinical studies, letters to the editor, animal studies, and studies with inappropriate comparator groups or outcome measures.

### 2.4. Data extraction, and assessment of the risk of bias

Following trial screening and quality assessment, 2 independent researchers extracted and summarized data on sensory and motor block characteristics, as well as adverse events associated with intrathecal anesthesia, with subsequent cross-verification performed to resolve any discrepancies. First, duplicate studies identified across different databases were removed using reference management software; next, irrelevant records were excluded via initial screening of titles and abstracts against the predefined eligibility criteria. The full texts of potentially eligible studies were then retrieved and carefully reviewed for definitive confirmation of inclusion. A standardized data extraction form was designed to collect key details from all finally included studies, and the synthesized information is presented in Table 1.

**Table 1:** The general characteristics of the enrolled studies.

Author, year, reference	Country	Sample size	Type of surgery	Intervention	ASA I/II/III	Time of surgery (min)
Sarma, 2015^[11]^	India	50	Lower limb surgery	5 µg dexmedetomidine with 15 mg bupivacaine	N/A	N/A
		50		50 µg clonidine with 15 mg bupivacaine	N/A	N/A
Kanazi, 2006^[12]^	Lebanon	16	Transurethral resection of prostate or bladder tumor	3 µg dexmedetomidine with 12 mg bupivacaine	3/9/4	56 ± 18
		16		30 µg clonidine with 12 mg bupivacaine	5/9/2	77 ± 48
Manohar, 2015^[13]^	India	25	Lower abdominal surgery	5 µg dexmedetomidine with 2.5 mL of 0.75% ropivacaine	19/6/0	54.80 ± 14.47
		25		50 µg clonidine with 2.5 mL of 0.75% ropivacaine	18/7/0	57.36 ± 11.78
Li, 2015^[14]^	China	21	Elective cesarean section	10 µg dexmedetomidine with 10 mg bupivacaine	20/1/0	43.31 ± 7.70
		21		75 µg clonidine with 10 mg bupivacaine	19/2/0	45.22 ± 7.95
Ganesh, 2018^[15]^	India	50	Lower abdominal surgery	3 µg dexmedetomidine with 17.5 mg bupivacaine	N/A	N/A
		50		30 µg clonidine with 17.5 mg bupivacaine	N/A	N/A
Manoharan, 2023^[16]^	India	30	Lower abdominal surgery	5 µg dexmedetomidine with 15 mg levobupivacaine	N/A	143.7 ± 30.2
		30		50 µg clonidine with 15 mg levobupivacaine	N/A	135.2 ± 15.4
Kishnani, 2025^[17]^	India	30	Lower limb orthopedic surgery	5 μg dexmedetomidine with 3.5 mL of 0.75% ropivacaine	N/A	N/A
		30		30 μg clonidine with 3.5 mL of 0.75% ropivacaine	N/A	N/A
Choudhary, 2023^[18]^	India	45	Hysterectomy	5 µg dexmedetomidine with 15 mg bupivacaine	33/12/0	N/A
		45		30 µg clonidine with 15 mg bupivacaine	33/12/0	N/A
Solanki, 2013^[19]^	India	30	Lower limb surgery	5 µg dexmedetomidine with 15 mg bupivacaine	26/4/0	119.5 ± 35.7
		30		50 µg clonidine with 15 mg bupivacaine	26/4/0	124.3 ± 34.1
Mahendru, 2013^[7]^	India	30	Lower limb surgery	5 µg dexmedetomidine with 12.5 mg mg bupivacaine	28/2/0	110.8 ± 33.7
		30		30 µg clonidine with 12.5 mg bupivacaine	26/4/0	99.8 ± 34.5

In accordance with the Cochrane risk of Bias Assessment Tool,^[20]^ 2 researchers (Meijuan Yang and Yu Zhang) evaluated the methodological quality of the included studies independently, covering the following domains: random sequence generation, allocation concealment, blinding of outcome assessment, incomplete outcome data, and selective reporting. Each domain was categorized as 1 of 3 risk levels: low risk of bias, high risk of bias, or unclear risk of bias (assigned when insufficient information was available to make a definitive judgment). Any discrepancies arising during the evaluation process were resolved through discussion and consultation with a 3rd researcher, thereby ensuring the objectivity and reliability of the assessment results.

### 2.5. Grading the quality of evidence

The Grading of Recommendations Assessment, Development, and Evaluation (GRADE) approach was employed to assess both the quality of the available evidence and the strength of the resulting recommendations in this review.^[21,22]^ The evidence quality was categorized into 4 levels – high, moderate, low, or very low – based on a comprehensive assessment of 5 key domains: risk of bias among included studies, inconsistency in study results, indirectness of evidence relative to the research question, imprecision of effect estimates, and potential publication bias. To ensure the standardization and rigor of the evaluation process, 2 independent reviewers conducted the GRADE assessment, with any discrepancies resolved through discussion or consultation with a 3rd adjudicator.

### 2.6. Statistical analysis

All statistical analyses were performed using Review Manager software (Version 5.3; The Cochrane Collaboration, Oxford, UK). For continuous data, the mean difference (MD) with 95% confidence interval (CI) was calculated; the standardized mean difference (SMD) was used for outcomes reported with different measurement units. Dichotomous outcomes were analyzed using the Mantel-Haenszel method. Heterogeneity across studies was evaluated using the *I*^2^ statistic. When significant heterogeneity was detected (defined as *I*^2^ > 50%), a sensitivity analysis was conducted by sequentially excluding each individual study, and a random-effects model was adopted; in the absence of significant heterogeneity (defined as *I*^2^ < 50%), a fixed-effects model was applied instead. When the number of included studies exceeded 10, Begg test and Egger test were performed to assess publication bias, using the metabias program (Version 1.2.4) in Stata/MP 12.0 for Windows (StataCorp LP, College Station). A *P* value < .05 was considered statistically significant.

## 3. Results

### 3.1. Literature screening and basic characteristics of enrolled studies

**Figure 1.**
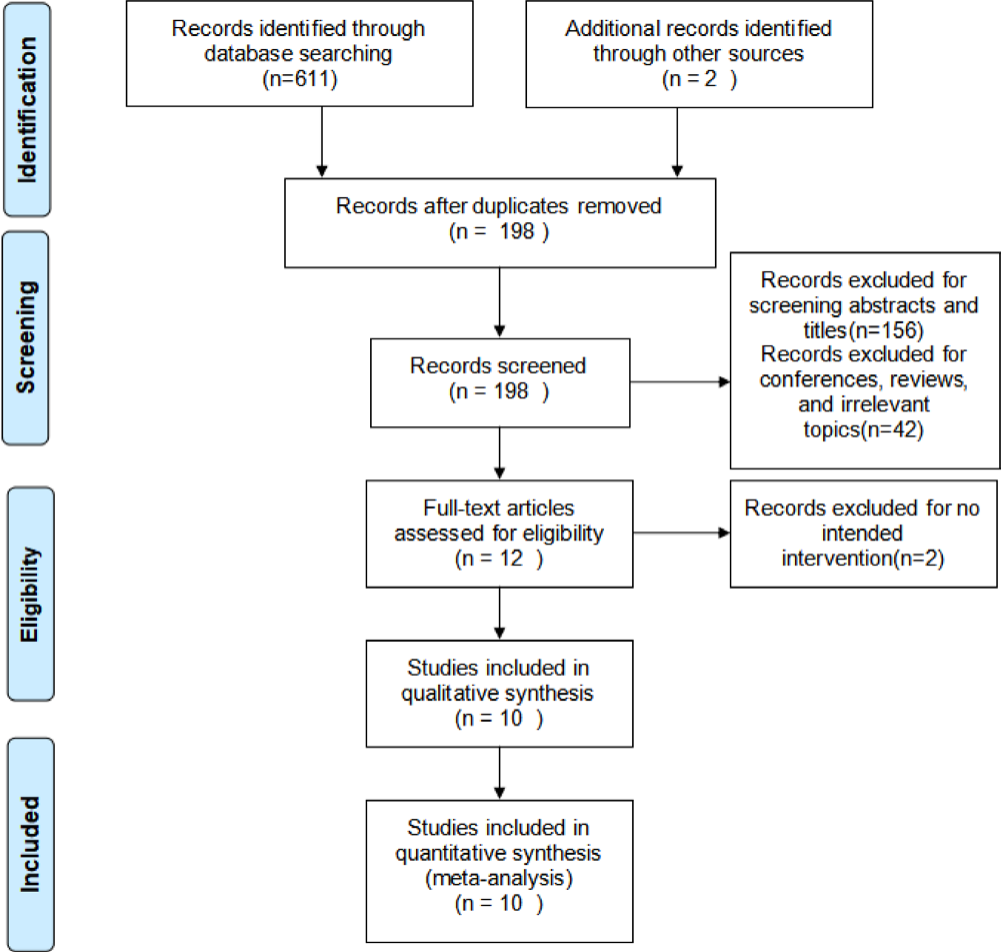
Flow chart of literature screening and the inclusion and exclusion processes.

The study selection flowchart is presented in Figure 1. Two independent reviewers 1st screened potential studies against the predefined inclusion and exclusion criteria. Any discrepancies arising during the screening process were resolved through group discussion. Ultimately, 10 studies involving 654 patients were included in this meta-analysis.

The basic information of all enrolled studies were shown in Table 1. The doses of dexmedetomidine and clonidine were different in each study. They were 5 µg dexmedetomidine plus 15 mg bupivacaine versus 50 µg clonidine plus 15 mg bupivacaine, 3 µg dexmedetomidine plus 12 mg bupivacaine versus 30 µg clonidine plus 12 mg bupivacaine, 5 µg dexmedetomidine plus 2.5 mL of 0.75% ropivacaine versus 50 µg clonidine plus 2.5 mL of 0.75% ropivacaine, 10 µg dexmedetomidine plus 10 mg bupivacaine versus 75 µg clonidine plus 10 mg bupivacaine, 3 µg dexmedetomidine plus 17.5 mg bupivacaine versus 30 µg clonidine plus 17.5 mg bupivacaine, 5 µg dexmedetomidine plus 15 mg levobupivacaine versus 50 µg clonidine plus 15 mg levobupivacaine, 5 μg dexmedetomidine plus 3.5 mL of 0.75% ropivacaine versus 30 μg clonidine plus 3.5 mL of 0.75% ropivacaine, 5 µg dexmedetomidine plus 15 mg bupivacaine versus 30 µg clonidine plus 15 mg bupivacaine, 5 µg dexmedetomidine plus 12.5 mg mg bupivacaine versus 30 µg clonidine plus 12.5 mg bupivacaine.^[7,11–19]^

### 3.2. Risk of bias assessment

**Figure 2.**
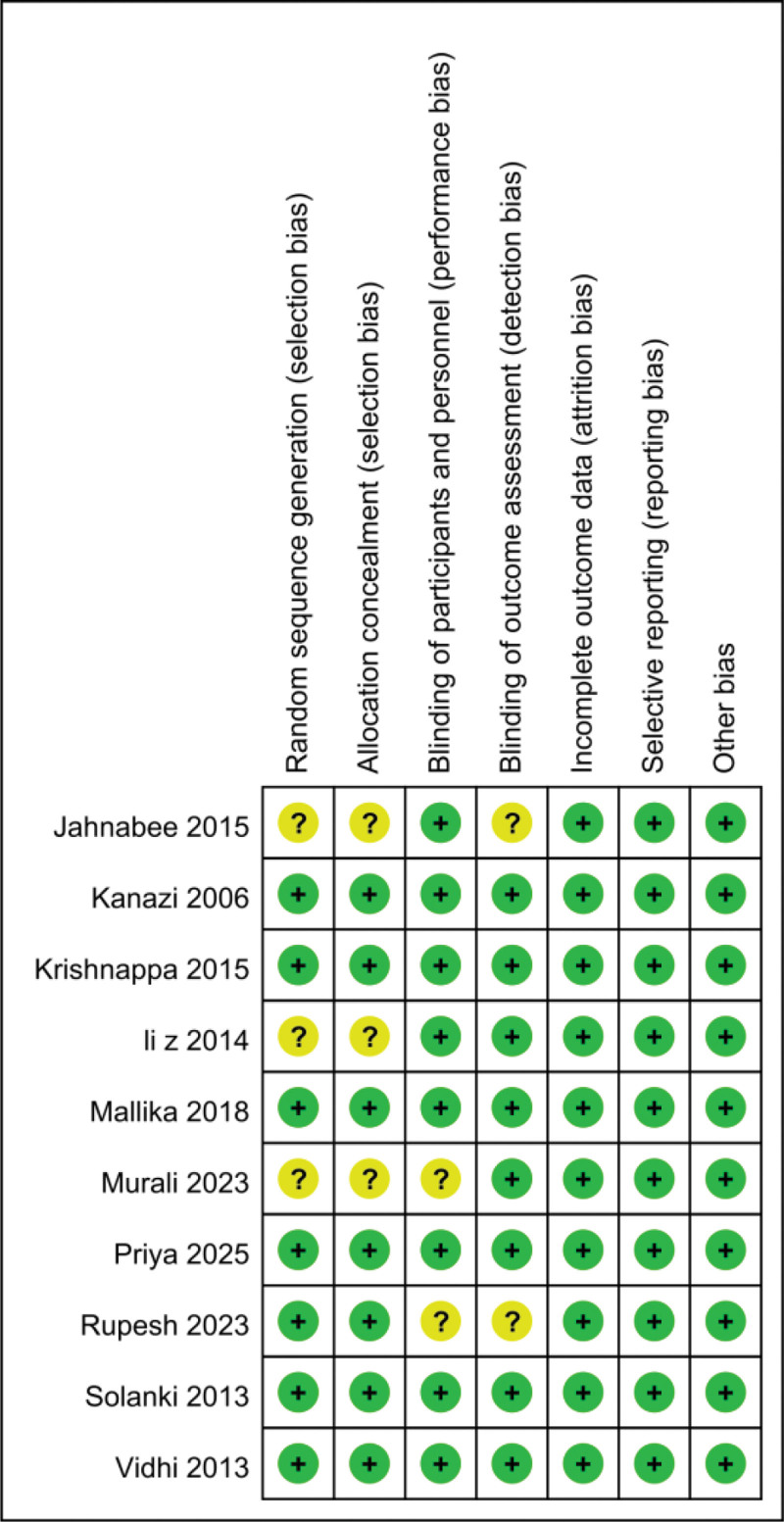
Risk of bias assessment of included studies. Green + dot, low risk of bias; yellow ? dot, unclear risk of bias.

The validity and quality of the enrolled RCTs focusing on intrathecal use of dexmedetomidine or clonidine were evaluated by using Cochrane Collaboration risk of bias tool. Among the results, 70% (7/10) studies had low risk in random sequence generation domain, only 30% (3/10) of the studies mentioned strategies for not mentioning allocation concealment, 80% (8/10) studies described blinding procedure of participants and personnel, and 80% (8/10) of the studies elaborated on the blinding of outcome assessment personnel. The details of risk of bias assessment were shown in Figure 2.

### 3.3. Characteristics of the block

#### 3.3.1. Onset of sensory block

The characteristics of spinal blockade are summarized in Table 2. Five studies involving 370 patients comparing dexmedetomidine with clonidine as local anesthetic adjuvants reported the onset time of sensory block. High heterogeneity was observed for this outcome (*I*^2^ = 80%), but sensitivity analysis failed to identify any single study as the definitive source of this heterogeneity. Therefore, a random-effects model was adopted for meta-analysis of this outcome. Based on the GRADE Summary of Findings table, the quality of evidence for sensory block onset time was rated as moderate (Table S1, Supplemental Digital Content, https://links.lww.com/MD/R557). Compared with the clonidine group, the dexmedetomidine group was associated with a faster onset of sensory block (SMD: −0.57, 95% CI: −1.06 to −0.090; *P* < .05).

**Table 2:** Comparison of characteristics of blockade between dexmedetomidine and clonidine group.

Characteristics of blockade	Number of studies (reference no.)	Number of patients in dexmedetomidine group	Number of patients in clonidine group	*I*^2^ (%)	Mean difference with (95% CI)	Std. mean difference with (95% CI)	*P* value
Onset of sensory block	5^[7,15,17–19]^	185	185	80	–	−0.57 (−1.06 to −0.09)	.02
Onset of motor block	7^[7,11,12,15,17–19]^	251	251	95	–	−0.52 (−1.39 to 0.36)	.25
Duration of sensory block	3^[7,14,17]^	81	81	0	29.87 (21.89–37.86)	–	<.00001
Duration of motor block	9^[7,11,12,14–19]^	302	302	89	31.12 (14.51–47.73)	–	.0002
Time to 1st rescue analgesia	5^[11,13–15,19]^	176	176	49	21.86 (13.14–30.59)	–	<.00001

#### 3.3.2. Onset of motor block

Data on the onset time of motor block were extracted from 7 studies involving 502 patients and are summarized in Table 2. An *I*^2^ value of 95% indicated substantial heterogeneity across these studies; however, sensitivity analysis failed to attribute this heterogeneity to any single study. Consequently, a random-effects model was adopted for meta-analysis of this outcome. Based on the GRADE Summary of Findings table, the quality of evidence for motor block onset time was rated as low (Table S1, Supplemental Digital Content, https://links.lww.com/MD/R557). Compared with the clonidine group, the dexmedetomidine group did not demonstrate a faster onset of motor block (SMD: −0.52, 95% CI: −1.39 to 0.36; *P* > .05).

#### 3.3.3. Duration of sensory block

Data on the duration of sensory block were extracted from 3 studies involving 162 patients and are summarized in Table 2. Heterogeneity across these studies was low (*I*^2^ = 0%), and thus a fixed-effects model was used for meta-analysis of this outcome. Based on the GRADE Summary of Findings table, the quality of evidence for sensory block duration was rated as moderate (Table S1, Supplemental Digital Content, https://links.lww.com/MD/R557). Compared with the clonidine group, the dexmedetomidine group demonstrated a longer duration of sensory block (MD = 29.87 minutes, 95% CI: 21.89–37.86; *P* < .05).

#### 3.3.4. Duration of motor block

Data on the duration of motor block were extracted from 9 studies involving 604 patients and are summarized in Table 2. High heterogeneity was observed across these studies (*I*^2^ = 89%), but sensitivity analysis failed to clearly attribute this heterogeneity to any single study. Therefore, a random-effects model was used for meta-analysis of this outcome. Based on the GRADE Summary of Findings table, the quality of evidence for motor block duration was rated as low (Table S1, Supplemental Digital Content, https://links.lww.com/MD/R557). Compared with the clonidine group, the dexmedetomidine group demonstrated a longer duration of motor block (MD = 31.12 minutes, 95% CI: 14.51–47.73; *P* < .05).

#### 3.3.5. Time to 1st rescue analgesia

Data on the time to 1st rescue analgesia (the time to 1st rescue analgesia was measured from the time of intrathecal injection to the time of 1st rescue analgesia) were extracted from 5 studies involving 352 patients and are summarized in Table 2. Heterogeneity across these studies was low (*I*^*2*^ = 49%), and a fixed-effects model was therefore used for meta-analysis of this outcome. Based on the GRADE Summary of Findings table, the quality of evidence for the time to 1st rescue analgesia was rated as low (Table S1, Supplemental Digital Content, https://links.lww.com/MD/R557). Compared with the clonidine group, the dexmedetomidine group was associated with a longer time to 1st rescue analgesia (MD = 21.86 minutes, 95% CI: 13.14–30.59; *P* < .05).

#### 3.3.6. Adverse effects

Adverse events including nausea and vomiting, bradycardia, hypotension, and shivering are summarized in Table 3. For all these adverse events, heterogeneity testing yielded an *I*^2^ value of 0% or 1%, and a fixed-effects model was therefore used for the meta-analysis. Based on the GRADE Summary of Findings table, the quality of evidence for most adverse events was rated as moderate (Table S1, Supplemental Digital Content, https://links.lww.com/MD/R557). No statistically significant differences were observed between the dexmedetomidine and clonidine groups with respect to the incidence of nausea and vomiting, bradycardia, hypotension, or shivering (*P* ≥ .05).

**Table 3:** Comparison of adverse effects between dexmedetomidine and clonidine group.

Adverse effects	Number of studies (reference no.)	Patients in dexmedetomidine group (incidence %)	Patients in clonidine group (incidence %)	*I*^2^ (%)	Risk ratio with (95% CI)	*P* value
Nausea and vomiting	9^[7,11–15,17–19]^	18/297 (6.06)	15/297 (5.05)	0	1.18 (0.64–2.18)	.59
Bradycardia	10^[7,11–19]^	30/327 (9.17)	25/327 (7.65)	0	1.20 (0.74–1.94)	.47
Hypotension	8^[7,11,13–18]^	54/281 (19.22)	52/281 (18.51)	1	1.04 (0.76–1.41)	.81
Shivering	6^[11,13–15,18,19]^	8/221 (3.62)	9/221 (4.07)	0	0.90 (0.38–2.15)	.81

## 4. Discussion

Our meta-analysis clearly demonstrated that, when used as an intrathecal adjuvant and compared with clonidine, dexmedetomidine yielded superior spinal block characteristics specifically, shortening the onset time of sensory block, prolonging the duration of both sensory and motor block, and extending the time to 1st rescue analgesia. No statistically significant differences were observed between the 2 drugs with respect to the incidence of nausea and vomiting, bradycardia, hypotension, or shivering.

The precise mechanism by which intrathecal α_2_-adrenoceptor agonists prolong the duration of sensory and motor blockade induced by local anesthetics has not been fully elucidated. This effect is not associated with alterations in the pharmacokinetic profile of local anesthetics, as clinical studies have demonstrated that co-administration of intrathecal dexmedetomidine with ropivacaine does not modify the plasma concentration – time curve of ropivacaine. This phenomenon is presumably attributable to complementary or synergistic interactions between the 2 classes of drugs, which act via distinct pharmacological pathways.^[23–25]^ Local anesthetics achieve nerve block by reversibly inhibiting voltage-gated sodium channels on nerve axons, thereby blocking the initiation and propagation of action potentials. In contrast, intrathecal α_2_-adrenoceptor agonists exert their effects by specifically binding to α_2_-adrenoceptors distributed on the presynaptic terminals of primary afferent C-fibers and postsynaptic neurons in the spinal dorsal horn.^[26–28]^

Moreover, a growing body of evidence has confirmed that the relative antinociceptive and block – prolonging potencies of intrathecal α_2_-adrenoceptor agonists are closely correlated with their binding affinities and selectivities for α_2_-adrenoceptors in the spinal dorsal horn. Compared with clonidine, dexmedetomidine not only exhibits a significantly higher binding affinity for spinal α_2_-adrenoceptors but also demonstrates a selectivity ratio for α_2_- over α_1_-adrenoceptors that is more than 100-fold greater.^[28–30]^ Therefore, the superior receptor selectivity and high binding affinity of dexmedetomidine may underpin its ability to yield a more rapid onset of sensory block and longer durations of both sensory and motor block when co-administered with intrathecal local anesthetics. Although only the 30-minute prolongation of sensory block duration observed in our study was statistically significant, its clinical relevance should be interpreted in the context of specific clinical scenarios. For instance, this 30-minute extension may confer clinical benefits in patients undergoing short-duration surgeries, where prolonged analgesia can reduce the requirement for additional analgesic medications.

Bradycardia and hypotension are widely recognized as the most prominent adverse events associated with intrathecal administration of α_2_-adrenoceptor agonists, as consistently documented in numerous relevant clinical studies and systematic reviews.^[31,32]^ Notably, in our meta-analysis, when dexmedetomidine and clonidine were used as adjuvants to local anesthetics via the intrathecal route, no statistically significant difference was observed in the incidence of these cardiovascular adverse events. A plausible explanation for this phenomenon is that standard doses of local anesthetics typically achieve near-maximal inhibition of sympathetic outflow within the spinal cord, which may mask potential differences in the sympathetic-modulating effects of the 2 α_2_-adrenoceptor agonists.^[28]^ Furthermore, a growing body of evidence suggests that the cardiovascular effects of intrathecal α_2_-adrenoceptor agonists are primarily dose-dependent rather than being determined by their receptor-binding affinity or specificity. Consequently, the addition of a low dose of α_2_-adrenoceptor agonists to intrathecal anesthetic regimens is unlikely to induce substantial hemodynamic fluctuations, and may even exert negligible effects on cardiovascular stability.

The incidence of shivering was low, and no significant difference in shivering rates was observed between the 2 groups, which might be due to the antishivering property of the α_2_ adrenergic agents. Clonidine and dexmedetomidine exert antipostoperative shivering effects primarily via dual mechanisms: inhibition of central thermoregulatory pathways and attenuation of the hyperadrenergic response triggered by perioperative stress.^[33]^ The precise etiology of perioperative nausea and vomiting has not yet been fully elucidated. A multitude of contributing factors are believed to be involved in its pathogenesis, including cerebral ischemia, sympathetic nerve blockade, and hypotension.^[34,35]^ Consistent with the shivering outcomes, we detected no significant difference in the incidence of nausea and vomiting between the clonidine and dexmedetomidine groups.

Several limitations of this meta-analysis should be considered when interpreting the results. First, a notable constraint of the present investigation relates to the predominantly low-to-moderate quality ratings assigned to outcomes via the GRADE system. This quality limitation can be primarily attributed to 3 methodological challenges: marked inconsistency (manifested as high heterogeneity) among the pooled studies, the fact that 30% (3/10) of the included studies did not report allocation concealment strategies, and imprecision arising from an inadequate number of reported events. Second, the number of included RCTs was relatively small – only 10 studies met the inclusion criteria and were incorporated into our analysis – which restricted the feasibility of conducting stratified subgroup analyses to explore potential sources of heterogeneity. Third, the included studies involved a variety of surgical procedures (including lower limb surgery, transurethral resection of prostate or bladder tumor, lower abdominal surgery, elective cesarean section, lower limb orthopedic surgery, and hysterectomy) and different local anesthetics (bupivacaine, levobupivacaine, and ropivacaine). These surgeries inherently vary in the degree of surgical trauma and pain pathway activation, which may help explain the high heterogeneity observed in this study. Additionally, the different doses of dexmedetomidine and clonidine used also exhibited substantial heterogeneity across the included studies. Fourth, because fewer than 10 studies contributed data for each outcome, Begg and Egger tests were not performed to assess publication bias. Moreover, our literature search was limited to peer-reviewed articles published in English-language databases; the exclusion of gray literature (such as unpublished trial reports or conference abstracts) may have introduced publication bias. Therefore, the overall risk of bias for the entire evidence base was high. This high level of overall bias risk indicates potential limitations in the internal validity of the included research. Accordingly, the conclusions drawn from the present meta-analysis should be interpreted with caution.

## 5. Conclusion

When used as an intrathecal adjuvant and compared with clonidine, dexmedetomidine yielded superior spinal block characteristics specifically, shortening the onset time of sensory block, prolonging the duration of both sensory and motor block, and extending the time to 1st rescue analgesia. No statistically significant differences were observed between the 2 groups with respect to the incidence of nausea and vomiting, bradycardia, hypotension, or shivering.

## Acknowledgments

We are sincerely grateful to all colleagues and peers who provided invaluable support and assistance during the entire process of this research.

## Supporting information

**Supporting table.** GRADE summary of findings table.

## Author contributions

**Conceptualization:** Meijuan Yang, Yu Zhang, Zhiyou Peng.

**Data curation:** Meijuan Yang, Yu Zhang, Zhiyou Peng.

**Formal analysis:** Meijuan Yang, Zhiyou Peng.

**Funding acquisition:** Meijuan Yang, Zhiyou Peng.

**Investigation:** Yu Zhang, Zhiyou Peng.

**Methodology:** Meijuan Yang, Yu Zhang, Zhiyou Peng.

**Project administration:** Zhiyou Peng.

**Resources:** Zhiyou Peng.

**Software:** Meijuan Yang, Zhiyou Peng.

**Supervision:** Zhiyou Peng.

**Validation:** Zhiyou Peng.

**Visualization:** Zhiyou Peng.

**Writing – original draft:** Zhiyou Peng.

**Writing – review & editing:** Zhiyou Peng.

## Supplementary Material


