# Supplementary material for: A comparative study of dexmedetomidine versus clonidine as additives for spinal anesthesia: A meta-analysis of clinical trials
Source: Medicine (Baltimore). 2026 Mar 20;105(12):e48102. doi: 10.1097/MD.0000000000048102 (PMC13008233; doi:10.1097/MD.0000000000048102)
Supplement: Supplementary file 1 [file medi-105-e48102-s001.doc]

**GRADE summary-of-findings (SoF) tables**

**onset of sensory block**

**
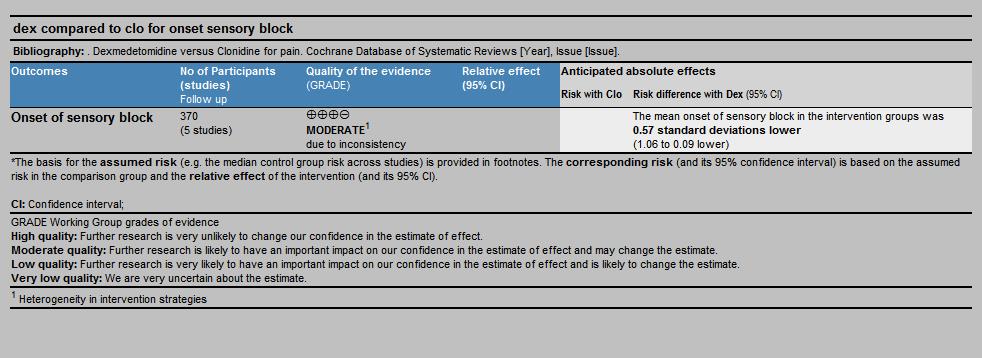
**

**onset of motor block**

**
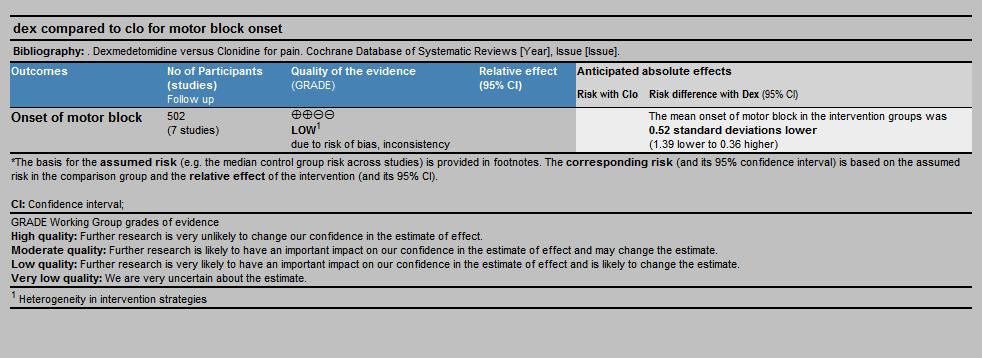
**

**duration of the sensory block**

**
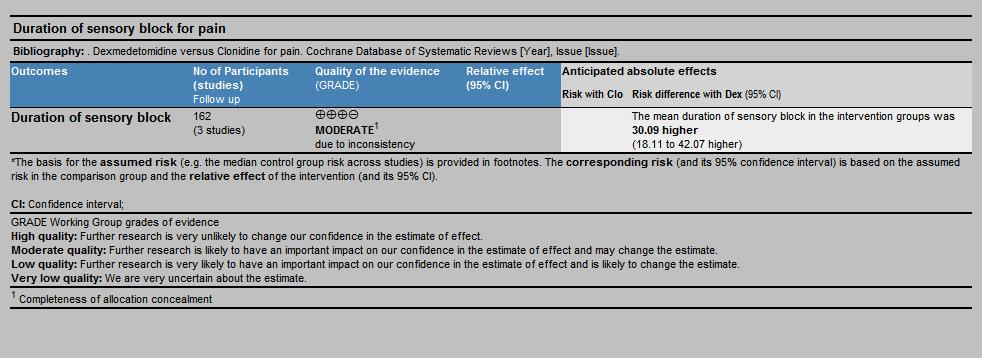
**

**duration of the motor block**

**
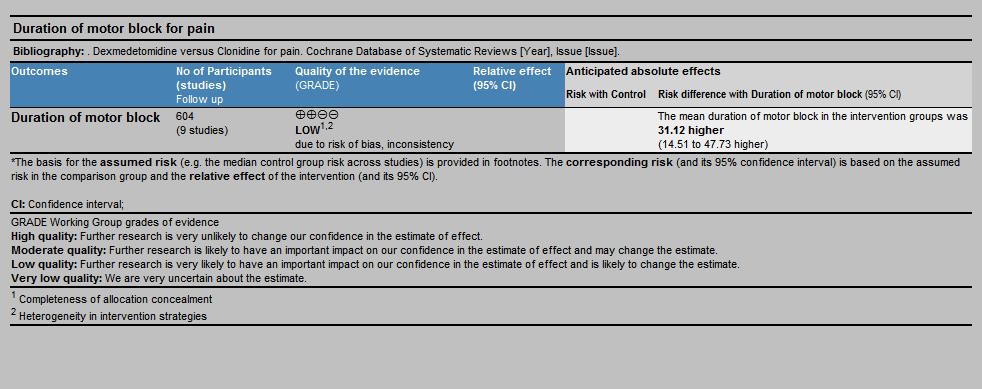
**

**time to first rescue analgesia**

**
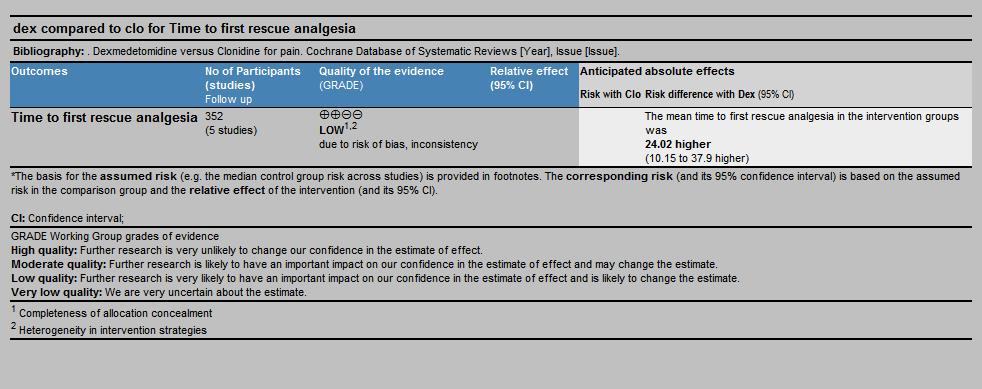
**

**Nausea vomiting**

**
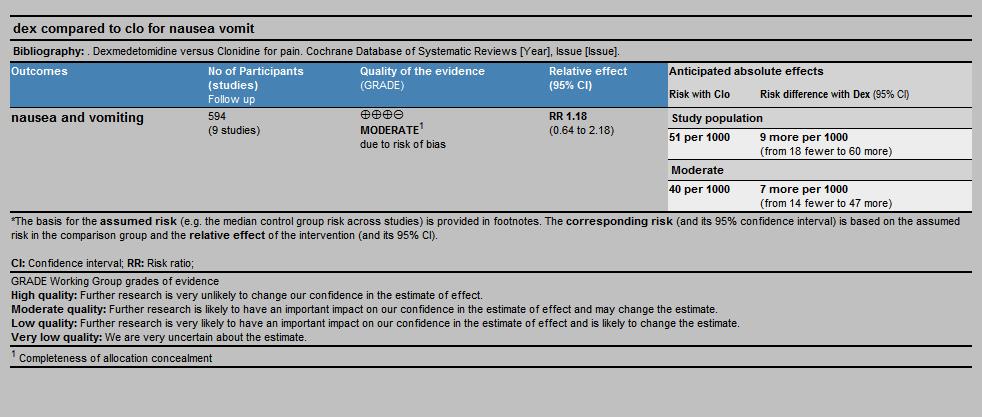
**

**Bradycardia**

**hypotension**

**
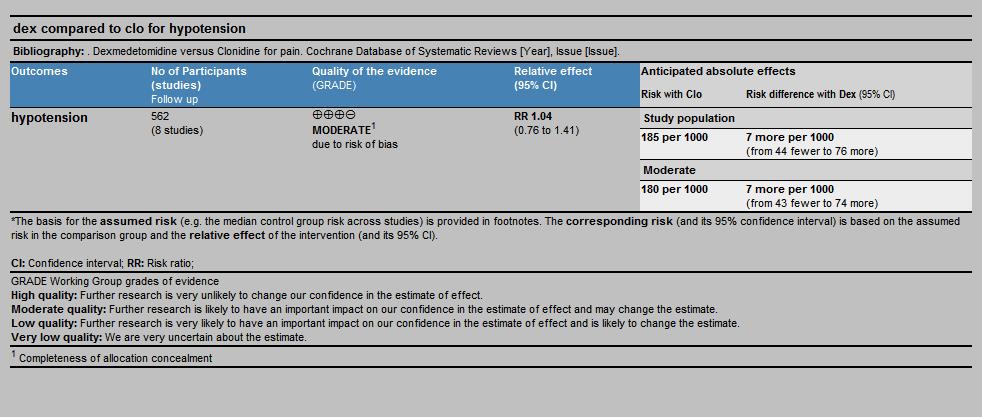
**

**shivering**

**
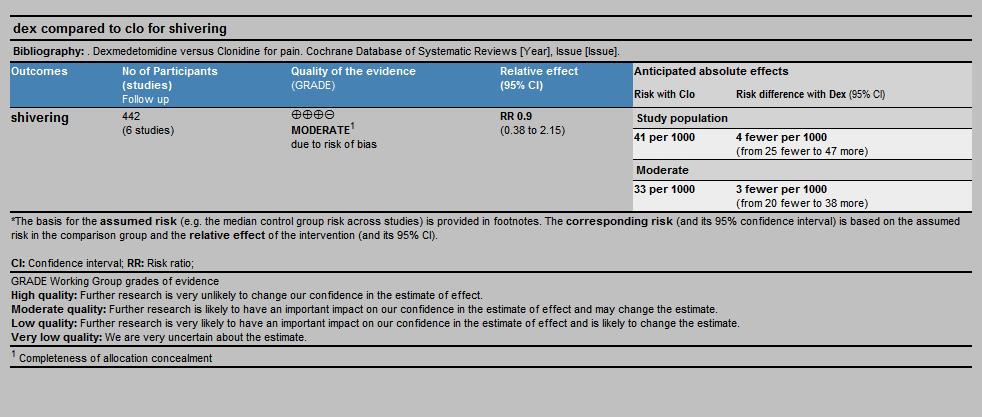
**
